# De novo acute lymphoblastic leukemia-like disease of high grade B-cell lymphoma with *MYC* and *BCL2* and/or *BCL6* rearrangements: a case report and literature review

**DOI:** 10.1186/s12907-017-0060-1

**Published:** 2017-11-09

**Authors:** Akiko Uchida, Yasushi Isobe, Yu Uemura, Yuji Nishio, Hirotaka Sakai, Masayuki Kato, Kaori Otsubo, Masahiro Hoshikawa, Masayuki Takagi, Ikuo Miura

**Affiliations:** 1 Division of Hematology & Oncology, Department of Internal Medicine, St. Marianna University School of Medicine, 2-16-1 Sugao, Miyamae-ku, Kawasaki, Kanagawa 216-8511 Japan; 2 Department of cytogenetics, SRL Diagnostics, Hachioji Laboratory, Tokyo, Japan; 3 Department of Pathology, St. Marianna University School of Medicine, Kawasaki, Japan

**Keywords:** High grade B-cell lymphoma with *MYC* and *BCL2* and/or *BCL6* rearrangements, Acute lymphoblastic leukemia-like disease, T(14;18)(q32;q21), *MYC*, *BCL2*

## Abstract

**Background:**

B-cell lymphomas harboring the 8q24/*MYC* plus 18q21/*BCL2* translocations are now referred to as high grade B-cell lymphoma with *MYC* and *BCL2* and/or *BCL6* rearrangements (HGBL-MBR). Although HGBL-MBR is frequently found in cases with diffuse large B-cell lymphoma or Burkitt lymphoma-like B-cell lymphoma, acute lymphoblastic leukemia (ALL)-like disease of HGBL-MBR (AL-HGBL-MBR) has been reported incidentally.

**Case presentation:**

A 69-year-old Japanese woman developed remittent fever and increasing systemic bone pain. The bone marrow examination revealed that more than 90% of nuclear cells were blastoid cells, which were positive for CD10, CD19, CD20, and surface IgMκ and negative for terminal deoxynucleotidyl transferase (TdT). Cytogenetic studies confirmed that the patient had de novo AL-HGBL-MBR with the extra copies of *MYC* and loss of chromosome 17p. She showed resistance to chemoimmunotherapy and died seven months after the diagnosis. The literature review identified further 47 de novo AL-HGBL-MBR cases within the last 32 years. The median age was 61 years (range, 27 − 86); the male/female ratio was 2.0. Thirty-eight cases (79%) presented a clinical picture of ALL at diagnosis; 14 (36%) of 39 available cases showed central nervous system involvement. Loss of 17p and translocations at 2p12–13, 3q27, 9p13 were frequently observed as additional cytogenetic abnormalities. Although the median survival of 46 available cases was only five months (range, 0.1–18), rituximab use significantly improved the survival of AL-HGBL-MBR (log-rank test, *P* = 0.0294).

**Conclusion:**

Our patient and most reported de novo AL-HGBL-MBR cases showed resistance to conventional chemoimmunotherapy and disastrous consequences. AL-HGBL-MBL is a rare, but should be considered a distinct clinical condition in HGBL-MBR. Other therapeutic strategies, such as using inhibitors of MYC and BCL2, are needed to overcome the chemoresistance of AL-HGBL-MBR.

## Background

Recurrent reciprocal chromosomal translocations have been observed in specific subtypes of B-cell lymphomas [1, 2]. Although the presence of t(8;14)(q24;q32) and t(14;18)(q32;q21) are hallmarks of Burkitt lymphoma (BL) and follicular lymphoma (FL), respectively, these translocations occur at different B-cell differentiation stages [1, 2]. The *IGH*-*BCL2* fusion resulting from t(14;18) is generated from the failure of VDJ recombination in the bone marrow (BM) at an early B-cell stage, whereas the *IGH*-*MYC* fusion resulting from t(8;14) almost always occurs as a consequence of the aberrant class-switch recombination in germinal centers (GCs) of lymphoid tissues [1–3].

B-cell lymphomas harboring concurrent translocations of 8q24/*MYC* mainly in combination with 18q21/*BCL2* are called “double-hit” lymphoma and now defined as “high grade B-cell lymphoma with *MYC* and *BCL2* and/or *BCL6* rearrangements (HGBL-MBR)” according to the current World Health Organization classification (WHO) of lymphoid neoplasms [2, 4]. HGBL-MBR is frequently found in diffuse large B-cell lymphoma (DLBCL) and BL-like B-cell lymphoma cases, which show poor prognosis when treated with standard regimen, R-CHOP (rituximab plus cyclophosphamide, doxorubicin, vincristine, and prednisone), with a median survival of around 12 months [5–7]. Although the WHO classification defines HGBL-MBR as the terminal deoxynucleotidyl transferase (TdT)-negative mature B cell neoplasm in spite of the cell morphology [4], several cases with acute lymphoblastic leukemia (ALL)-like disease of HGBL-MBR (AL-HGBL-MBR) have been reported incidentally [8–33]. AL-HGBL-MBR is clinically characterized as the acute onset disease with the initial manifestation of BM infiltration by blastoid B cells but lacks obvious tumors, suggestive of primary lymphoma lesions. However, the characteristics have not been fully elucidated. We herein present an AL-HGBL-MBR case and conducted a literature review using PubMed to clarify the feature of this disease.

## Case presentation

**Fig. 1:**
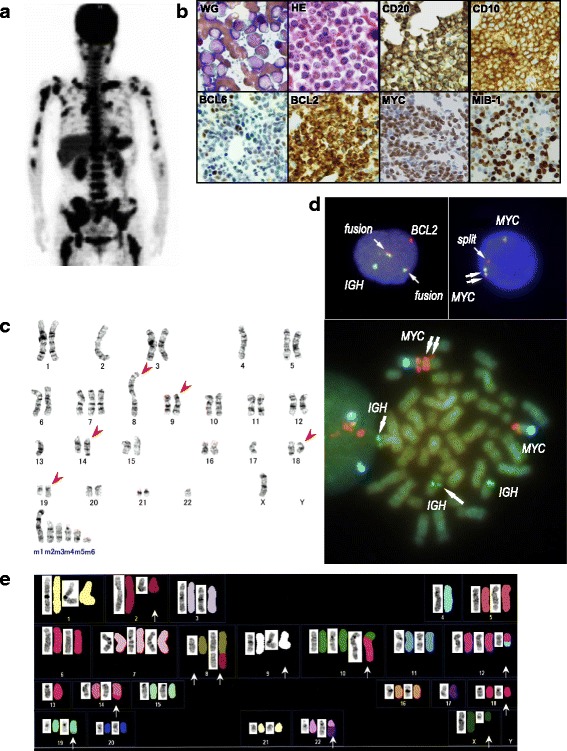
Radiological, cytological, histological, immunophenotypic, and cytogenetic findings of AL-HGBL. **a** F-18-fluorodeoxyglucose (FDG) positron emission tomography detected the strong accumulation of FDG in the liver, spleen, and whole-body bone areas. **b** Bone marrow preparations stained with Wright-Giemsa (WG) and hematoxylin-eosin (HE) detected sheets of blastoid cells with fine chromatin and only a few vacuoles. Leukemic cells were strongly positive for CD20, CD10, and BCL2, and weakly positive for BCL6. **c** The karyotype of bone marrow cells was examined using G-banding. Red arrowheads indicate the derivative chromosomes. **d** The FISH analysis of interphase cells confirmed that t(14;18)(q32;q21) resulted in fusion between *IGH* (green) and *BCL2* (red) and also that one *MYC* split signal (red) was located beside the two amplified *MYC* genes. In addition, the FISH analysis of metaphase cells indicated the amplification of *MYC* (red) at 8q24 in derivative chromosome 8 and did not fuse to *IGH* (green). White arrows indicate these aberrations. **e** SKY revealed that 8q24 and 19q13.1 were translocated to chromosomes 2 and 11, respectively. In addition, the loss of chromosome 17p was confirmed because derivative chromosome 22 contained chromosome 17q. White arrows indicate the derivative chromosomes and marker chromosomes detected by G-banding

The condition of a 69-year-old Japanese woman was good until she developed remittent fever for one week. She had no previous history of lymphoma and presented to our institution with fever and increasing systemic bone pain. A physical examination showed no lymphadenopathy or hepatosplenomegaly. Laboratory tests showed a white blood cell count of 4.7 × 10^9^/L, hemoglobin level of 119 g/L, platelet count of 104 × 10^9^/L, and lactate dehydrogenase (LDH) level of 12,623 IU/L. A peripheral blood smear revealed leukoerythroblastosis with 7.5% blastoid cells. F-18-fluorodeoxyglucose (FDG) positron emission tomography (PET) detected the relatively strong accumulation of FDG in the liver, spleen, vertebrae, and bilateral clavicles, humeri, ilia, and femora (maximum standardized uptake value (SUVmax) 4.8~13.0) (Fig. 1a). A BM examination revealed that more than 90% of nuclear cells were medium-sized blastoid cells with fine chromatin (Fig. 1b). A flow cytometric analysis showed that the cells were positive for CD10, CD19, CD20, HLA-DR, and surface IgMκ, but were negative for CD3, CD5, CD13, CD33, CD34, and TdT. The patient was tentatively diagnosed with mature B-cell leukemia and admitted to our hospital. She received R-hyper CVAD/MA (rituximab plus cyclophosphamide, vincristine, doxorubicin, dexamethasone/methotrexate, and cytarabine). Although her serum LDH levels decreased to approximately 1000 IU/L after two courses of the intensive regimen, blastoid cells remained in the BM. Therefore, we changed the regimen to dose-adjusted EPOCH-R (rituximab plus etoposide, prednisolone, vincristine, cyclophosphamide, and doxorubicin). After two courses of dose-adjusted EPOCH-R, leukemic cells remained and lost the expression of CD20. She died seven months after the diagnosis because of disease progression.

## Histological and immunohistochemical analyses on BM specimens

We morphologically reviewed BM specimens using hematoxylin and eosin (HE) staining. Immunohistochemistry (IHC) was also performed on formalin-fixed, paraffin-embedded sections. The monoclonal antibodies used for IHC were CD10 (56C6) (Nichirei, Tokyo, Japan), CD20 (L26) (Dako, Glostrup, Denmark), BCL2 (124) (Dako), BCL6 (P1F6) (Nichirei), MIB-1 (Dako), and MYC (Y60) (Abcam, Cambridge, UK). The MIB-1 index was calculated as the percentage of MIB-1-stained nuclei, which were counted among total 500 nuclei in three different visual fields.

The BM was massively infiltrated by medium-sized blastoid cells with fine chromatin and inconspicuous nucleoli (Fig. 1b). There were no paratrabecular lymphocyte aggregates, which are typically observed in FL [9, 11]. The blasts were strongly positive for CD20, CD10, BCL2, and the MYC protein and weakly positive for BCL6, indicating a GC B-cell phenotype (Fig. 1b). The MIB-1 index was unexpectedly low, at approximately 60% (Fig. 1b). This case was not diagnosed with DLBCL and suspected to have AL-HGBL-MBR.

## Cytogenetic and fluorescence in situ hybridization studies

Standard G-banding and fluorescence in situ hybridization (FISH) analyses were performed. The probes used for the FISH analysis were a Vysis® LSI® *IGH*/*BCL2* dual-color, dual-fusion translocation probe, Vysis® LSI® *IGH*/*MYC*/CEP8 tri-color dual fusion probe, and Vysis® LSI® *MYC* dual-color breakapart rearrangement probe (Abbott Molecular, Des Plaines, IL). The FISH analysis was applied to interphase and metaphase cells. Spectral karyotyping (SKY) was also performed for the aid of the interpretation of G-banded metaphase cells.

A G-banding analysis of BM specimens detected a complex karyotype including t(14;18)(q32;q21) and add(8)(q24) (Fig. 1c). A chromosomal segment of an unknown origin was translocated to the telomeric side of 8q24. The karyotype was 46,X,-X,-2,-4,+7,-8,add(8)(q24),inv(9)(p12q13),-13,t(14;18)(q32;q21),-17,add(19)(q13.1),-22,+mar1,+mar2,+mar3,+mar4,+mar5,+mar6 [18] (Fig. 1c). The FISH analysis of interphase and metaphase cells confirmed the fusion between *IGH* and *BCL2* and the split and extra copies of *MYC* at 8q24 (Fig. 1d). Furthermore, SKY identified that 8q24 and 19q13.1 were translocated to chromosomes 2 and 11, respectively. G-banding and SKY confirmed the loss of chromosome 17p (Fig. 1c, e). Taken together, this case was diagnosed with AL-HGBL-MBR with the extra copies of *MYC* and loss of chromosome 17p.

## Literature review

We selected the cases with BM involvement (at least ≥20% of nuclear cells) of non-centroblastic, non-immunoblastic TdT-negative blastoid B cells harboring both t(14;18)(q32;q21) and 8q24/*MYC* translocations, which was confirmed by G-banding and/or FISH analyses. The cases with either of them were excluded. Although secondary AL-HGBL-MBR arising from FL was also found, we selected only de novo cases to evaluate the survival from diagnosis because the onset and outcome were clearly recorded in each report.

**Fig. 2:**
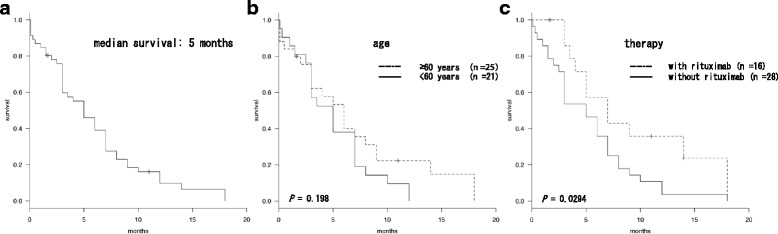
Survival duration of the collected de novo AL-HGBL-MBR cases. **a** Kaplan-Meier survival distributions of 46 cases. Median survival was five months [95% CI 3–7]. **b** There was no significant difference in the survival durations between older (≥60 years, *n* = 25) and younger (<60 years, *n* = 21) patients (median five [95% CI 3–7] vs. six [95% CI 3–9] months, log-rank test, *P* = 0.198). **c** Among 44 cases receiving chemotherapy, the survival in patients treated with rituximab (*n* = 16) was significantly improved, compared with in patients treated without rituximab (*n* = 28) (median seven [95% CI 3.5-NA] vs. five [95% CI 3–7] months, log-rank test, *P* = 0.0294). All *P* values were two-tailed, and *P* < 0.05 was considered significant

We have identified further 47 de novo AL-HGBL-MBR cases from the published data (Table 1).[8–33] The median age at presentation is 61 years (range, 27–86); the male/female ratio was 2.0. Although 10 cases (21%) had a modest mass lesion at extranodal sites, the rest showed the clinical picture of ALL. In addition, 14 (36%) of 39 available cases showed central nervous system involvement. Unlike BL, L2-type morphology according to the French-American-British classification was observed in 14 (33%) of 42 available cases (Table 1). The immunophenotype of leukemic cells was positive for CD10, CD19, and CD20 in most cases (Table 1). Cytogenetic studies showed that loss of 17p and translocations at 2p12–13, 3q27, 9p13 are frequently observed as additional chromosomal aberrations, and at least nine (20%) cases had loss of 17p (Table 1). Most patients reported prior to 2003 received standard chemotherapy for ALL, whereas patients reported since 2003 were frequently treated with rituximab-combined chemotherapy for lymphomas (Table 1). Nevertheless, 42 patients (88%) died because of disease progression at the time of publication. Median survival of 46 cases, the observation period of which is well documented, was only five months (range 0.1–18 months) from the diagnosis. Their survival distribution was illustrated in Fig. 2a. The survival curves were determined using the Kaplan-Meier method. All analyses were performed using EZR (Saitama Medical Center, Jichi Medical University, ver.1.33) [34]. There was no significant difference in survival between older (≥60 years, *n* = 25) and younger (<60 years, *n* = 21) patients (log-rank test, *P* = 0.198) (Fig. 2b). Among the examined 46 cases, 44 cases received chemotherapy, and their survival durations were significantly improved by rituximab use (log-rank test, *P* = 0.0294) (Fig. 2c).

**Table 1:** Clinicopathologic features of published cases with de novo acute lymphoblastic leukemia-like disease of high grade B-cell lymphoma with *MYC* and *BCL2* and/or *BCL6* rearrangements

Case (ref. n)	Age/sex	Extramedullary lesion at presentation	CNS	FAB criteria	Immunophenotype				Main cytogenetic abnormalities	FISH or gene analysis		Curative therapy	Survival
					CD10	CD19	CD20	sIg		*IGH-BCL2*	*MYC*		
1 (8)	57/F	bone	+	L3	+	+	–	ND	t(8;22)(q24;q12^a^),t(14;18)(q32;q21)	NA	NA	ALL regimen	7 mo
2 (9)	74/M	palate, L (cervical, pretracheal)	–	L3	NA	+	NA	μ	t(8;22)(q24;q11),t(14;18)(q32;q21)	NA	NA	ALL regimen	3 mo
3 (9)	37/M	none	–	L2	–	+	+	IgMλ	t(8;22)(q24;q11),t(14;18)(q32;q21)	NA	NA	ALL regimen	12 mo
4 (9)	73/M	none	+	L3	+	+	+	IgMκ	t(8;22)(q24;q11),t(14;18)(q32;q21)	NA	NA	ALL regimen	8 mo
5 (10)	62/M	H, S	–	L3	+	+	+	ND	t(8;14)(q24;q32),t(14;18)(q32;q21)	NA	NA	ALL regimen	3 mo
6 (11)	35/M	none	–	L3	+	+	NA	ND	t(14;18)(q32;q21)	GR (+)	GR (+)	ALL regimen	0.3 mo
7 (12)	27/M	L (IP, RP)	+	L3	+	+	–	ND	t(8;22)(q24;q11),der(14)t(14;18)(q32;q21)	NA	NA	ALL regimen	5 mo
8 (13)	67/M	none	–	L3	NA	+	+	IgGκ	t(8;22)(q24;q11),t(14;18)(q32;q21)	NA	NA	none	0.1 mo
9 (14)	71/M	none	–	L3	−/+	+	+	IgMλ	t(1;3;11)(q42.3;q27.1;q23.1),der(8)t(8;9)(q24.2;p13.3),t(14;18)(q32.3;q21.3),der(17)t(17;?)(p13;?)	GR (+)	GR (+)	none	0.1 mo
10 (15)	36/F	IP mass	–	NA	+	+	NA	ND	t(8;22)(q24;q11),t(14;18)(q32;q21)	NA	GR (−)	ALL regimen	3 mo
11 (15)	60 M	none	+	L2	NA	NA	NA	NA	t(8;22)(q24;q11),t(14;18)(q32;q21),+der(14)t(14;18)(q32;q21)	NA	GR (−)	ALL regimen	6 mo
12 (16)	40/M	GL, paravertebral mass	–	L2	+	+	+	NA	der(6)t(6;8)(q1?;q24),add(8)(q24),der(9)t(8;9)(q24;p1?),t(14;18)(q32;q21),del(17)(p11)	NA	NA	ALL regimen	1 mo
13 (17)	69/M	H	–	L3	–	+	+	IgMκ	der(7;17)(q10;q10),+der(8)t(8;14;18)(q24;q32;q22),add(14)(q32),del(18)(q21)	GR (+)	NA	ALL regimen	5 mo
14 (18)	41/F	none	–	L3	+	+	+	NA	t(2;3)(p12;q27),del(8)(q24),t(14;18)(q32;q21)	GR (+)	GR (+)	ALL regimen	10 mo
15 (19)	50/F	GL	–	L2	+	+	+	IgMκ	t(3;4)(q27;p13),t(8;14;18)(q24;q32;q21),+ider(8)(q10)t(8;14;18)(q24;q32;q21)	GR (+)	GR (+)	ALL regimen	0.1 mo
16 (19)	44/M	GL, S	+	NA	+	+	+	IgMκ	t(3;13)(q27;q14),t(8;22)(q24;q11),t(14;18)(q32;q21),+der(18)t(14;18)(q32;q21)	GR (+)	GR (−)	NCVBP, IVAM, ASCT	7 mo
17 (19)	46/F	GL, S, Asc, PE	+	L2	+	+	+	IgMκ	t(2;3)(p12;q27),add(8)(q24),der(14)t(8;14)(q24;q32),der(18)t(14;18)(q32;q21)	GR (+)	GR (+)	ACVBP, allo-SCT	3 mo
18 (20)	62/F	none	NA	L3	NA	NA	NA	NA	t(2;8)(p12;q24),t(14;18)(q32;q21)	NA	NA	none	0.1 mo
19 (21)	48/M	GL	–	L2	NA	NA	NA	NA	t(8;9)(q24,p13),t(14;18)(q32;q21)	NA	NA	R-CHOP	3.5 mo
20 (22)	72/M	H, S	–	L2	+	NA	+	IgGκ	t(8;9)(q24;p13),t(14;18)(q32;q21)	NA	NA	R-EPOCH	4 mo
21 (23)	71/M	S (mild)	–	L2	–	+	+	γ	t(1;2)(q22–23;p13),t(8;14)(q24;q32),t(14;18)(q32;q22)	GR (+)	GR (+)	ALL regimen	2 mo
22 (24)	50/F	L (axillary), SC mass	–	L3	+	+	NA	ND	t(2;3)(p12;q27),t(8;22)(q24;q11),t(14;18)(q32;q21),-17	fusion (+)	split (+)	ALL regimen	7 mo
23 (25)	29/M	none	–	L3	+	+	+	NA	+8,t(8;22)(q24;q11),t(14;18)(q32;q21)	NA	split (+)	R-CHOP, R-ICE, R-hyper-CVAD/MA	5 mo
24 (25)	72/M	none	–	L3	+	+	+	NA	t(8;22)(q24;q11.2),t(14;18)(q32;q21)	NA	NA	R-hyper-CVAD/MA	11 mo^b^
25 (25)	50/F	none	+	L3	+	+	+	NA	t(8;22)(q24;q11),t(14;18)(q32;q21)	fusion (+)	split (+)	R-hyper-CVAD/MA	3 mo
26 (25)	32/M	L (mesenteric)	+	L3	+	+	+	NA	t(1;3)(p32;q26.2),t(8;22)(q24;q11),add(14)(q32),t(14;18)(q32;q21)	fusion (+)	split (+)	hyper-CVAD/MA	8 mo
27 (25)	67/M	L (P), small intestine	+	L3	+	+	+	NA	t(8;14)(q24;q32),der(8)t(8;14)t(14;18)(q32;q21),der(14)t(8;14),+add(14)(q32),i(17)(q10)	fusion (+)	split (+)	R-hyper-CVAD/MA, velcade	9 mo
28 (25)	61/M	L (RP), colon, prostate	+	L3	NA	NA	NA	NA	t(8;22)(q24;q11),t(14;18)(q32;q21),+der(14)t(14;18)	fusion (+)	NA	hyper-CVAD/MA, MOAP	9 mo
29 (25)	42/F	small intestine, omentum, breast	–	L3	+	+	+	NA	t(8;14)(q24;q32),t(14;18)(q32;q21),der(17)t(10;17)(q22;q10)	fusion (+)	NA	proMACECytaBOM, CHOP, ESHAP, hyper-CVAD/MA, SCT, RT	12 mo
30 (25)	63/M	testis, lip	–	L3	+	+	+	NA	NA	fusion (+)	split (+)	hyper-CVAD	18 mo
31 (26)	57/F	none	NA	NA	+	+	–	NA	der(3)t(3;14;?)(q27;q32;?),t(8;14)(q24;q32),der(18)t(14;18)(q32;q21)	fusion (+)	split (+)	CODOX-M/IVAC	2.5 mo
32 (26)	60/M	none	NA	L3	+	+	+	D	t(2;8)(p12;q24),der(8)t(2;8)(p12;q24),t(14;18)(q32;q21),?i(17)(q10)	fusion (+)	split (+)	hyper-CVAD, CODOX-M	3 mo
33 (26)	63/M	GL, S (mild)	NA	L3	+	+	+	D	der(3)t(1;3)(q23;q27),t(8;22)(q24;q11),t(14;18)(q32;q21)	fusion (+)	split (+)	CODOX-M/IVAC	6 mo
34 (26)	76/F	none	NA	L3	–	+	NA	D	t(8;22)(q24,q11),t(14;18)(q32;q21)	fusion (+)	split (+)	VAD	6 mo
35 (26)	59/F	none	NA	L2	+	NA	NA	NA	t(8;9)(q24;p13),t(14;18)(q32;q21)	fusion (+)	split (+)	ALL regimen	1.5 mo
36 (26)	69/M	L (IP), H	NA	L3	+	+	+	D	t(14;18)(q32;q21)	fusion (+)	split (+)	ALL regimen	1.5 mo
37 (26)	86/F	none	NA	L2	+	+	+	ND	add(9)(p13),t(14;18)(q32;q21)	fusion (+)	split (+)	none	0.5 mo
38 (27)	43/F	pancreas	+	L3	+	+	+	IgMλ	t(8;14)(q24;q32),t(14;18)(q32;q21)	fusion (+)	split (+)	R-CODOX-M/IVAC	5 mo
39 (28)	61/M	GL, S, PE	–	L3	+	+	+	γ	t(3;5)(q27;q15),t(8;14;18)(q24;q32;q21),+der(8)t(8;14;18),+der(18)t(8;14;18)	fusion (+)	split (+)	R-hyper-CVAD,	NA^c^
40 (29)	42/M	L (IP), S	+	L2	+	+	+	κ	der(8)del(8)(q12.1q12.3)del(8)(q24.21q24.21)t(8;12)(q24.21;p12.1),der(12)del(12)(p12.1p12.1)t(8;12)(q24.21;p12.1),t(14;18)(q32;q21)	fusion (+)	split (+)	R-CHOP, ALL regimen	7 mo
41 (30)	72/M	bone, liver	+	NA	+	NA	+	NA	t(3;8)(q27;q24),t(14;18)(q32;q21)	fusion (+)	split (+)	ALL regimen	NA^c^
42 (31)	64/M	L (NA)	–	NA	+	NA	+	NA	NA	fusion (+)	split (+)	R-EPOCH	1.7 mo^c^
43 (31)	72/M	L (NA)	–	NA	+	NA	+	NA	t(3;22)(q27;q11.2),t(8;14)(q24;q32),t(14;18)(q32;q21)	fusion (+)	split (+)	R-EPOCH	1.6 mo^c^
44 (32)	74/F	none	+	L2	+	+	+	κ	–8,del(11)(q23q25),del(13)(q12q14),t(14;18)(q32;q21),+18	fusion (+)	split (+)	R-CHOP	3 mo
45 (32)	67/M	colon	–	L3	+	+	−/+	κ	NA	fusion (+)	split (+)	R-CHOP	11 mo^d^
46 (32)	71/M	L (mediastinal)	–	L3	+	+	+	κ	der(8)t(8;14;18)(q24;q32;q21),der(14)t(8;14)(q24.1;q32),der(18)t(14;18)(q32;q21)	fusion (+)	split (+)	R-EPOCH, R-ICE, ASCT	14 mo
47 (33)	60/M	GL	NA	L2	+	+	+	λ	+8,inv(8)(p11.2q24)×2,t(14;18)(q32;q21),-17	fusion (+)	split (+)	R-hyperCVAD, ofatumumab + EPOCH	18 mo
48 (present case)	69/F	none	–	L2	+	+	+	IgMκ	add(8)(q24),t(14;18)(q32;q21),-17	fusion (+)	split (+), EC (+)	R-hyper-CVAD/MA, R-EPOCH	7 mo

*Abbreviations*: *Ref n* reference number, *FAB criteria* blastoid cell morphology according to the French-American-British classification, *CNS* development of central nervous system involvement, *sIg* surface immunoglobulin, *M* male, *F* female, *L* lymphadenopathy, *H* hepatomegaly, *S* splenomegaly, *SC* subcutaneous, *IP* intraperitoneal, *RP* retroperitoneal, *GL* generalized lymphadenopathy, *Asc* ascites, *PE* pleural effusion, *P* pelvic, *ND* not detected, *D* detected, *NA* not available, *GR* gene rearrangement, *EC* extra copies, *ALL regimen* multidrug chemotherapy for acute lymphoblastic leukemia, *NCVBP* mitoxantrone, cyclophosphamide, vinblastine, bleomycin, and prednisone, *IVAM* ifosfamide, etoposide, cytarabine, and methotrexate, *ASCT* autologous stem cell transplantation, *ACVBP* doxorubicin, cyclophosphamide, vinblastine, bleomycin, and prednisone, *allo-SCT* allogeneic stem cell transplantation, *R* rituximab, *CHOP* cyclophosphamide, doxorubicin, vincristine, and prednisone, *EPOCH* etoposide, prednisone, vincristine, cyclophosphamide, and doxorubicin, *ICE* ifosfamide, carboplatin, and etoposide, *hyper-CVAD/MA* cyclophosphamide, vincristine, doxorubicin, dexamethasone alternating with methotrexate and cytarabine, *MOAP* methotrexate, vincristine, asparaginase, and prednisone, *proMACEcytaBOM* prednisone, vincristine, methotrexate, doxorubicin, cyclophosphamide, etoposide, cytarabine, and bleomycin, *ESHAP* etoposide, methylprednisolone, cytarabine, and cisplatin, *RT* radiation therapy, *CODOX-M/IVAC* cyclophosphamide, vincristine, doxorubicin, methotrexate alternating with ifosfamide, etoposide, and cytarabine, *VAD* vincristine, doxorubicin, and dexamethasone, *POMP* prednisone, vincristine, methotrexate, and mercaptopurine

^a^This description is according to original work [8]

^b^Alive (disease status was not described)

^c^Alive with disease at the time of publication

^d^Alive with no evidence of disease at the time of publication

## Discussion

Based on the previous history and histopathology, our case was diagnosed with de novo AL-HGBL-MBR. Bone pain at the initial presentation was likely caused by rapid cell proliferation and the sudden onset of the disease in the BM. The literature review revealed that around 80% of de novo AL-HGBL-MBL cases had a typical clinical picture of ALL, and the features of present case were consistent with those of ALL. The noticeable behavior of this case is the unresponsiveness to R-hyper CVAD/MA and dose-adjusted EPOCH-R, which are expected to be effective regimens for not only BL but also HGBL-MBR [7, 35]. Indeed, DLBCL-type HGBL-MBR is reported to have a better prognosis than that of BL-like HGBL-MBR, which seems to have common clinical features with AL-HGBL-MBR [36]. The differences of cell morphology in HGBL-MBR may influence the therapeutic efficacy of the conventional regimens. The median survival of the collected cases suggests that AL-HGBL-MBR may show the most unfavorable prognosis in any type of B-cell lymphomas. Despite the disastrous consequence, the present review showed that rituximab use had a positive impact on survival in HGBL-MBR cases.

t(14;18)-harboring FL arising from GC B cells usually presents an indolent clinical behavior, while this type of lymphoma often undergoes clonal evolution.[37] AL-HGBL-MBR also develops from FL, and the clinical features of secondary AL-HGBL-MBR are nearly the same as those of de novo cases [9, 11, 15, 32] The prognosis of secondary AL-HGBL-MBR is likewise very poor [9, 11, 15, 32]. Besides alterations in *MYC*, disruptions in p53 and p14^ARF^, and an additional 3q27/*BCL6* translocation are considered to be the dominant changes in transformed FL [37]. These alterations overlap with those of de novo AL-HGBL-MBR cases [9, 11, 15, 32] Although these abnormalities may incrementally accumulate during disease progression in FL, our case suggest that t(14;18)-carrying B cells suddenly develop into AL-HGBL-MBR when they have simultaneously acquired gene rearrangements and the amplification of *MYC* as well as the loss of 17p at the BM. If not, t(14;18)-carrying B cells may develop into a common FL. Even though this acquisition is a temporally distinct event, the accumulation of additional genetic abnormalities in t(14;18)-carrying B cells may eventually develop AL-HGBL. This situation is similar to that of ALL with *BCR*/*ABL1*. ALL with *BCR*/*ABL1* develops primarily and also arises from the blast crisis of chronic myelogenous leukemia. In AL-HGBL-MBR, the first hit is t(14;18), while the aggressive nature may be provided by further aberrations including the deregulation of *MYC* and disruption of p53. Therefore, FISH analyses regarding *MYC* and *TP53* should be evaluated, when HGBL-MBR is suspected, in t(14;18)-harboring neoplasms.

The leukemic cells in the present case strongly expressed the MYC protein, which precludes entry into the G0 phase. However, many of them remained in the G0 phase, in which the MIB-1 antibody failed to stain the nucleus [38, 39]. This condition indicates that the G0/G1 switch regulation was also impaired in the present case. In spite of the overexpression of MYC, MIB-1 index in HGBL-MBR cases was reported to vary from 25% to 100% [40, 41]. The suboptimal response to the R-hyper CVAD/MA and EPOCH-R regimens may be explained by the existence of MIB-1-negative and BCL2-positive cells. Recent in vitro studies suggested that concurrent inhibition of BCL2 and MYC may have therapeutic potential for the treatment of AL-HGBL-MBR patients [42, 43]. This strategy may overcome the resistance to conventional chemoimmunotherapy.

## Conclusions

Our case and a review of the literature indicate that de novo AL-HGBL-MBR is a rare but may be a distinct clinical condition in HGBL-MBR. AL-HGBL-MBR may be the most aggressive disease among all t(14;18)-harboring neoplasms. Because conventional chemotherapeutic regimens are ineffective, other therapeutic strategies, such as using inhibitors against BCL2 and MYC, may elicit the potential to overcome the chemoresistance. The further accumulation of molecular evidences to illustrate AL-HGBL-MBR is needed.

## Abbreviations

AL-HGBL-MBR: ALL-like disease of HGBL-MBR
ALL: Acute lymphoblastic leukemia
BL: Burkitt lymphoma
BM: Bone marrow
DLBCL: Diffuse large B-cell lymphoma
EPOCH-R: Etoposide, prednisolone, vincristine, cyclophosphamide, doxorubicin, and rituximab
FDG: F-18-fluorodeoxyglucose
FISH: Fluorescence in situ hybridization
FL: Follicular lymphoma
GC: Germinal center
HE: Hematoxylin and eosin
HGBL-MBR: High grade B-cell lymphoma with *MYC* and *BCL2* and/or *BCL6* rearrangements
IHC: Immunohistochemistry
LDH: Lactate dehydrogenase
PET: Positron emission tomography
R-CHOP: Rituximab, cyclophosphamide, doxorubicin, vincristine, and prednisone
R-hyper CVAD/MA: rituximab, cyclophosphamide, vincristine, doxorubicin, and dexamethasone/methotrexate and cytarabine
SKY: Spectral karyotyping
SUVmax: Maximum standardized uptake value
TdT: Terminal deoxynucleotidyl transferase
WHO: World Health Organization classification
